# Supplementary material for: Human centromere repositioning activates transcription and opens chromatin fibre structure
Source: Nat Commun. 2022 Sep 24;13:5609. doi: 10.1038/s41467-022-33426-2 (PMC9509383; doi:10.1038/s41467-022-33426-2)
Supplement: Supplementary file 1 — Supplementary Information [file 41467_2022_33426_MOESM1_ESM.pdf]

Human centromere repositioning activates transcription and opens chromatin fibre structure

Catherine Naughton, Covadonga Huidobro, Claudia R. Catacchio, Adam Buckle, Graeme R. Grimes, Ryu-Suke Nozawa, Stefania Purgato, Mariano Rocchi, Nick Gilbert

Supplementary material

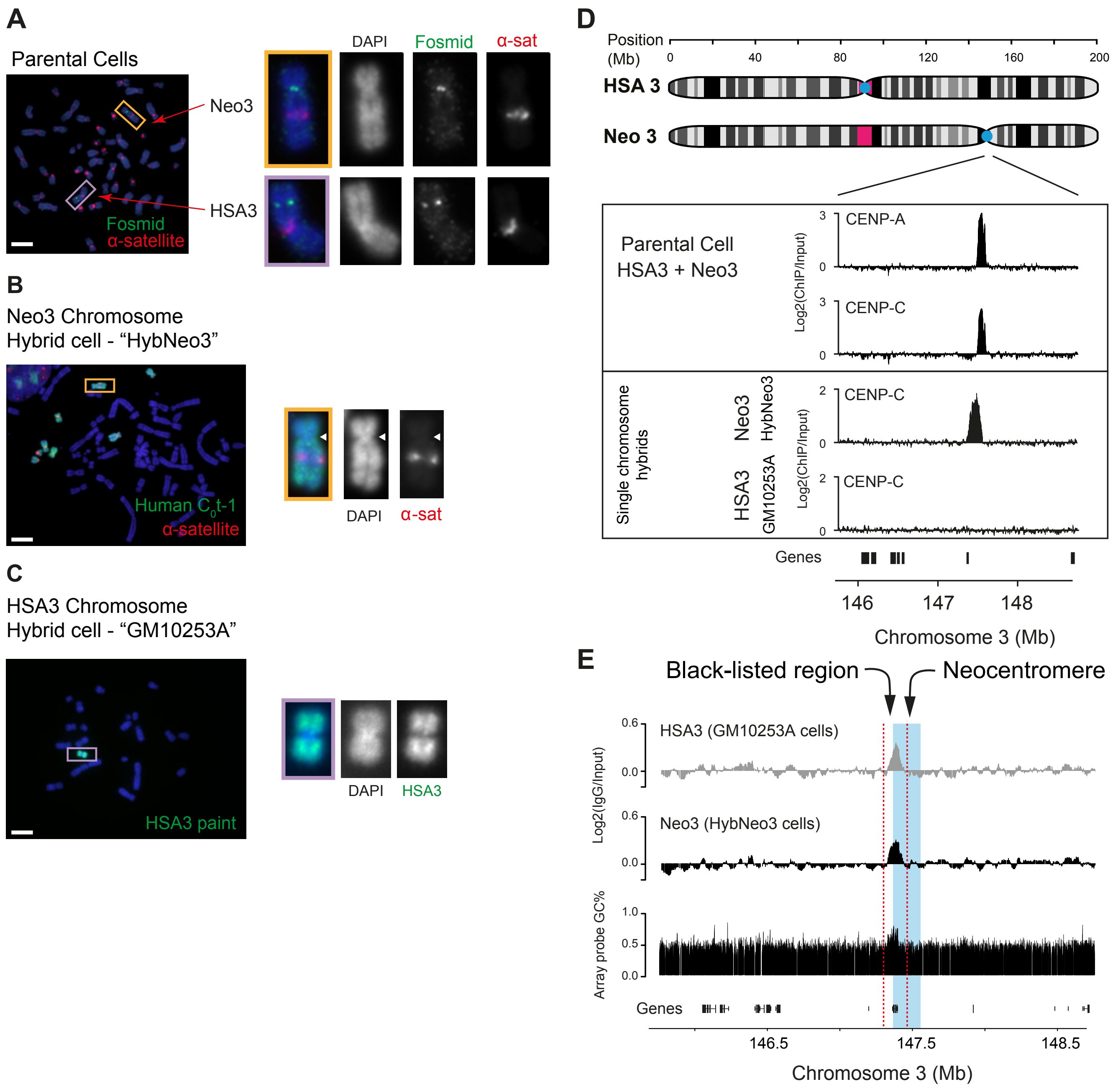

Supplementary Fig. 1. Cell line model system used to interrogate centromeric chromatin structure and mapping of neocentromere to 3q24 (relates to Figure 1).
(A) Parental human lymphoblastoid cells with one canonical chromosome 3 (HSA3, purple frame) and one chromosome 3 with the centromere relocated to form a neocentromere at 3q24 (Neo3, orange frame). Representative image of DNA FISH with **α**-satellite specific probe (red) and a 3q24 fosmid probe (green) (n = 2 biologically independent experiments with 30 metaphase spreads analysed per experiment). Bar is 5 μm. **(B)** The Neo3 chromosome was retained after fusion of the parental cell line with a hamster cell to create a hybrid line called “HybNeo3”. Representative image of metaphase spread of HybNeo3 hybridized with human C0t-1 DNA (green) identifying the eight human chromosomes present in this human/hamster hybrid (4,6,8,11,13,18, X and Neo3 (orange frame)) and a human **α**-satellite specific FISH probe (red) (n = 2 biologically independent experiments with 30 metaphase spreads analysed per experiment). Bar is 5 μm. **(C)** The GM10253A hybrid cell line has a single canonical human chromosome 3 (HSA3). Representative image of metaphase spread of GM10253A hybridized with a human chromosome 3 paint (green; purple frame) (n = 2 biologically independent experiments with 30 metaphase spreads analysed per experiment). Nuclei are counterstained with DAPI. Bar is 5 μm. **(D)** Top, ideogram depicting chromosomes HSA3 and Neo3. Bottom, distribution of CENP-A and CENP-C ChIP signal in parental and hybrid cells at 3q24. **(E)** Top, signal for control IgG ChIP-chip and bottom microarray probe GC composition (%). Blacklisted region (chr3: 147324413-147482213; hg38) is marked by red dashed lines. Neocentromere core (chr3: 147400413-147591023) defined from CENP-C (panel A) is marked in blue.

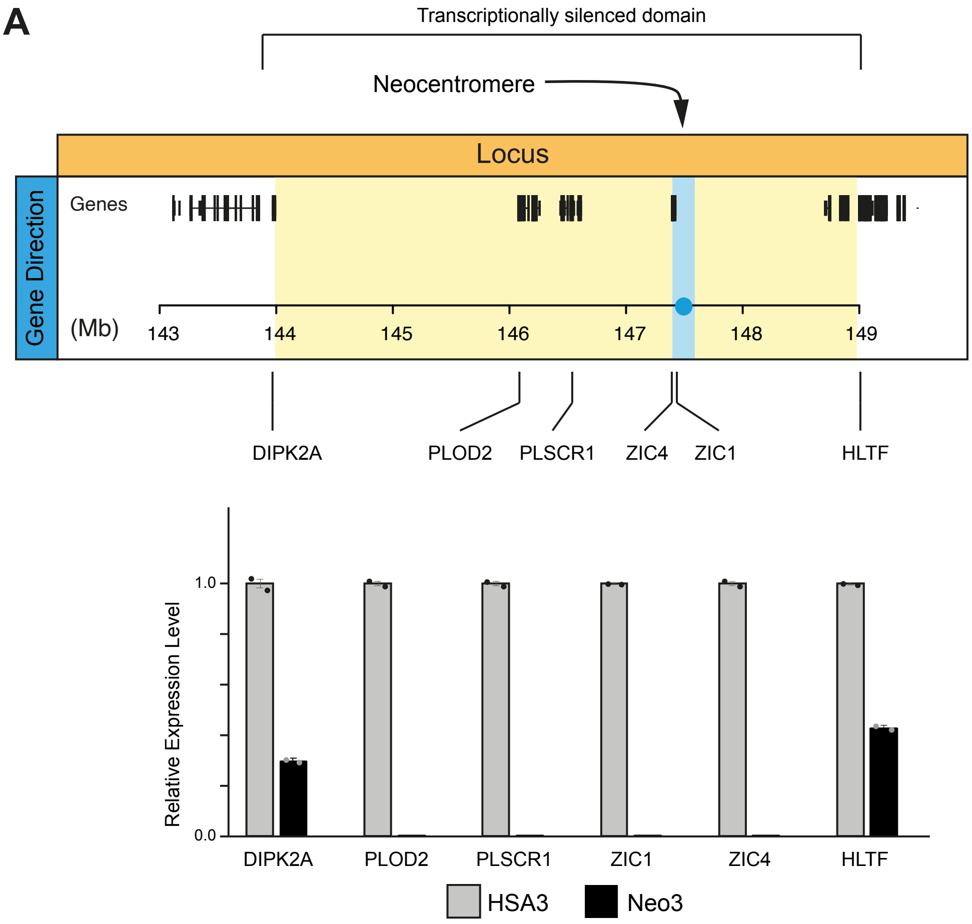

Supplementary Fig. 2. Transcription repression at the pericentromeric domain (Relates to Figure 2) Top, diagram showing individual genes at 3q24, yellow block corresponds to pericentromere domain, blue is the neocentromere. Bottom, RT-qPCR expression data for genes within and bordering the pericentromeric heterochromatic domain, showing expression from HSA3 and gene silencing on Neo3 (n = 2 per primer pair). Data are presented as mean values +/- SD. Supplementary table 2 contains primer sequences. Source data are provided as a Source Data file.

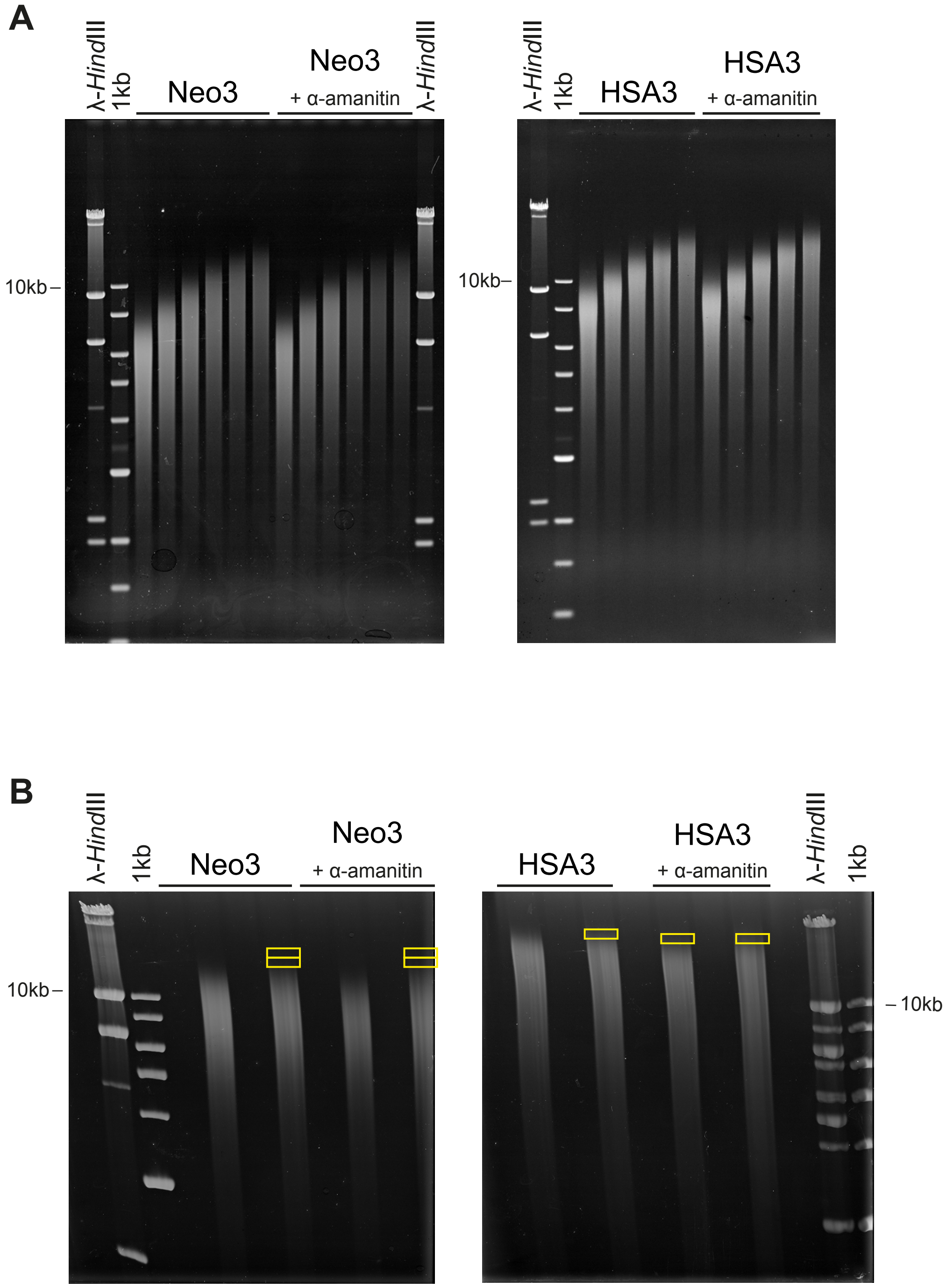

Supplemenary Fig. 3. Isolation of "open" chromatin probes (Relates to Figure 4).
Soluble chromatin isolated from hybrid cell nuclei by micrococcal digestion was fractionated by size and structure on a sucrose gradient. **(A)** Representative image of agarose gel electrophoresis of DNA purified from sucrose gradient fractions (n = 2 biologically independent experiments). **(B)** DNA from gradient fractions was size selected by PFGE. Representative image, n = 2 biologically independent experiments. DNA fragments ≈10 kb longer than the bulk of the DNA signal, corresponding to "open" or disrupted chromatin, was purified from gel slices (yellow boxes).

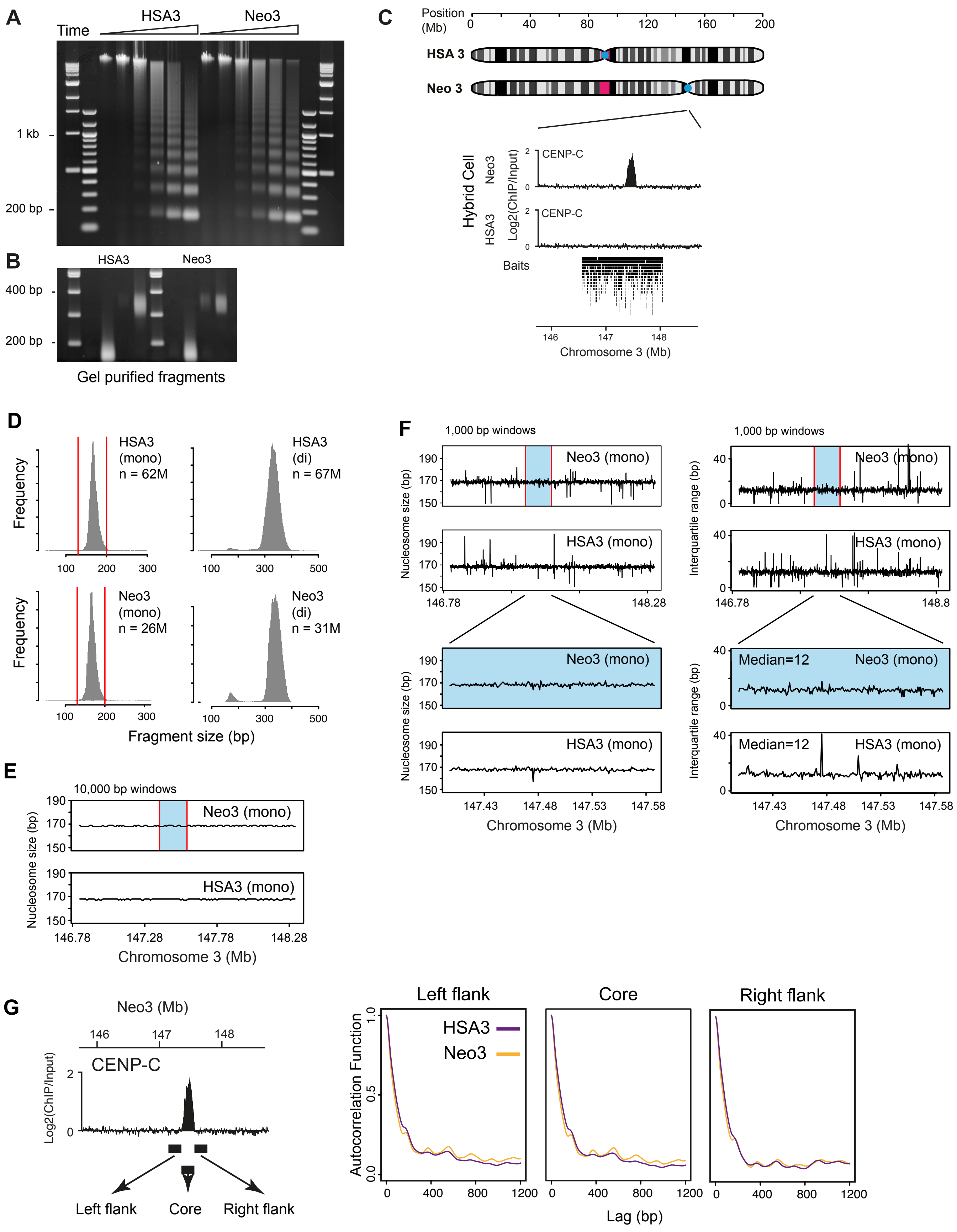

Supplementary Fig. 4
Legend over page

Supplementary Fig. 4. Arrangement of nucleosomes around neocentromere (Relates to Figure 5). (A) Agarose gel electrophoresis of DFF digested nuclei isolated from HSA3 and Neo3 containing cells. Mono and di-nucleosome fragments were excised, and the DNA extracted using β-agarase. Representative image, n = 4 biologically independent experiments. **(B)** Agarose gel electrophoresis of purified mono- and di-nucleosomes fragments used for nucleosome mapping. Representative image, n = 4 biologically independent experiments. **(C)** Top, ideogram depicting HSA3 and Neo3 chromosomes with enlargement of 3 Mb region around the neocentromere (marked by CENP-C). Bottom, genomic location of the capture baits used to enrich for 1.5 Mb of neocentromeric region. **(D)** Size distribution of mono and di nucleosomes (bp) isolated from HSA3 and Neo3 cells for 1.5 Mb around the region corresponding to the neocentromere. **(E)** Mono nucleosome size distribution in 10 kb windows across the 1.5 Mb captured domain (neocentromere marked in blue). **(F)** Left, mono nucleosome size (median) in 1 kb windows across the 1.5 Mb captured domain and focussed region covering the neocentromere (marked in blue). Right, variance (interquartile range) in mono nucleosome size in 1 kb windows across the 1.5 Mb captured domain and focussed region covering the neocentromere (marked in blue). **(G)** Autocorrelation of nucleosome dyad coverage at left flank, centromere core and right flank for different lag (bp).

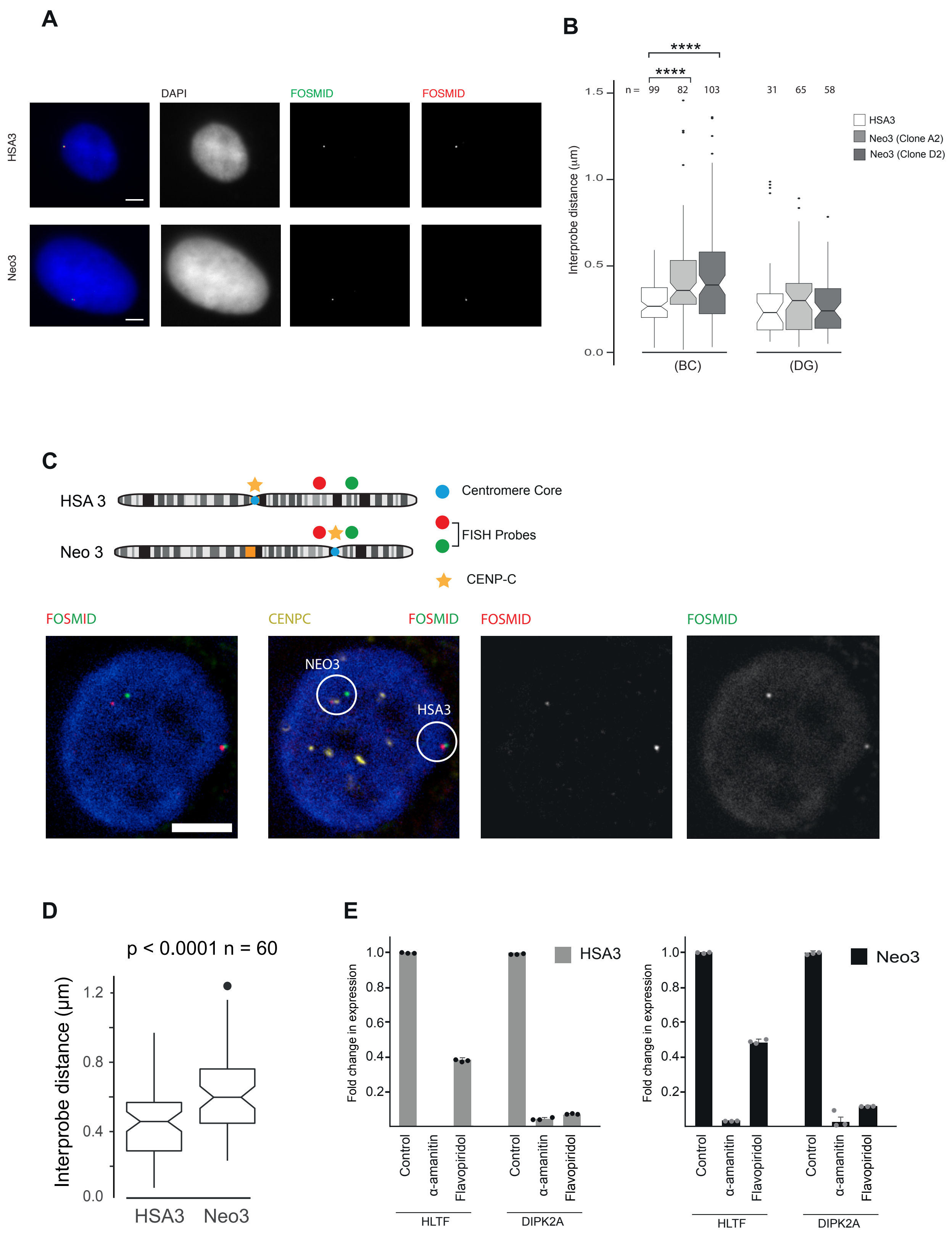

Supplementary Fig. 5
Legend over page

Supplementary Fig. 5. Large scale chromatin is decompacted at the neocentromere in the parental cell line (Relates to Figure 6).

(A) Representative images of 3D-FISH on HSA3 and Neo3 chromosomes hybridized to probe B (red) and C (green) in single chromosome human-hamster hybrid nuclei, counterstained with DAPI (n = 2 biologically independent experiments). Bar is 5 μm. **(B)** Boxplot showing interprobe distance measurements (μm) for the pairs of fosmid probes indicated in HSA3 (white) and Neo3 (grey) chromosomes in GM10253A and two additional clones (A2 and D2) of the human-hamster hybrid expressing the 3q24 neocentromere. Exact p values for a Wilcoxon test (two-sided) were (BC: HSA3 and Neo3(Clone A2)) 2.923e-05 and (BC: HSA3 and Neo3(Clone D2)) 2.856e-05. **(C)** Top, chromosome 3 ideogram indicating CENP-C immunofluoresence signal (yellow) and the neocentromere specific FISH probes (green and red). Bottom right, representative image of 4 colour 3D immuno-FISH for identifying the chromosome 3 harbouring a neocentromere at 3q24 due to the presence of the CENP-C signal proximal to one pair of fosmid probes (n = 2 biologically independent experiments). Below left, three colour representation of the same image used for measuring interprobe distance. Bar is 5 μm. **(D)** Boxplot showing interprobe distance measurements (μm) between the pair of fosmid probes (B and C, see Fig 6A) for the HSA3 and Neo3 chromosomes in the parental lymphoblastoid cells. Exact p value for a Wilcoxon test (two-sided) was 2.305e-05. **(E)** RT-qPCR expression data for genes in the pericentromere region flanking the neocentromere domain in cells carrying the HSA3 and Neo3 chromosomes, following transcription inhibition with **α**-amanitin (5h) or flavopiridol (3h). This data (n = 3) is one representative experiment, data from a second independent biological replicate can be found in the source data file. Data are presented as mean values +/- SD. B and E Data are shown as boxplots with median (middle line), 25th–75th percentiles (box), and min-max values (whiskers), p-values are for a Wilcoxon test (two-sided); *, $p < 0.05$; **, $p < 0.01$; ***, $p < 0.001$; ****, $p < 0.0001$. Source data are provided as a Source Data file.

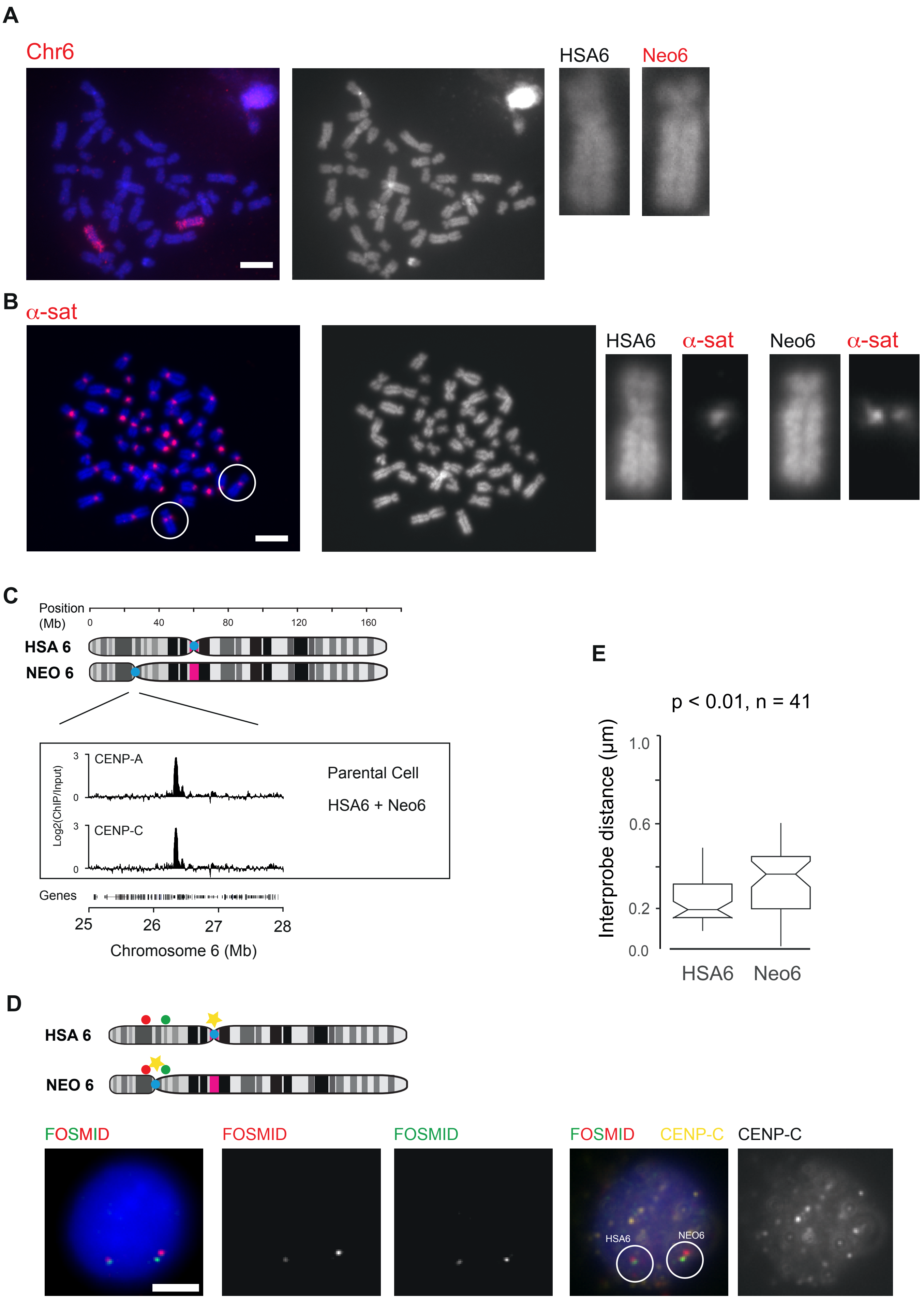

Supplementary Fig. 6
Legend over page

Supplementary Fig. 6. Large scale chromatin is decompacted at another human neocentromere on chromosome 6 (Relates to Figure 6).

Parental human lymphoblastoid cells with one canonical chromosome 6 (HSA6) and one chromosome 6 with the centromere relocated to form a neocentromere at 6p22.2 (Neo6). **(A)** Representative images of DNA FISH with a human chromosome 6 specific paint (red), (n = 2 biologically independent experiments with 30 metaphase spreads analysed per experiment). Bar is 5μm. **(B)** Representative images of DNA FISH with **α**-satellite specific probe (red) (n = 2 biologically independent experiments with 30 metaphase spreads analysed per experiment). Bar is 5μm. **(C)** Top, ideogram depicting chromosomes HSA6 and Neo6. Bottom, distribution of CENP-A and CENP-C ChIP signal in parental cells at 6p22.2. **(D)** Top, chromosome 6 ideogram indicating CENP-C immunofluoresence signal (yellow) and the neocentromere 6 specific FISH probes A and C (green and red). Bottom right, representative image of 4 colour 3D immuno-FISH for identifying the chromosome 6 harbouring a neocentromere at 6p22.2 due to the presence of the CENP-C signal proximal to one pair of fosmid probes. Below left, three colour representation of the same image used for measuring interprobe distance. Bar is 5μm. **(E)** Boxplot showing interprobe distance measurements (μm) between the pair of fosmid probes for the HSA6 and Neo6 chromosomes in the parental lymphoblastoid cells. Exact p value for a Wilcoxon test (two-sided) was 0.002461. Data are shown as boxplots with median (middle line), 25th–75th percentiles (box), and min-max values (whiskers), p-values are for a Wilcoxon test (two-sided); *, $p < 0.05$; **, $p < 0.01$; ***, $p < 0.001$; ****, $p < 0.0001$. Bar is 5μm. Source data are provided as a Source Data file.

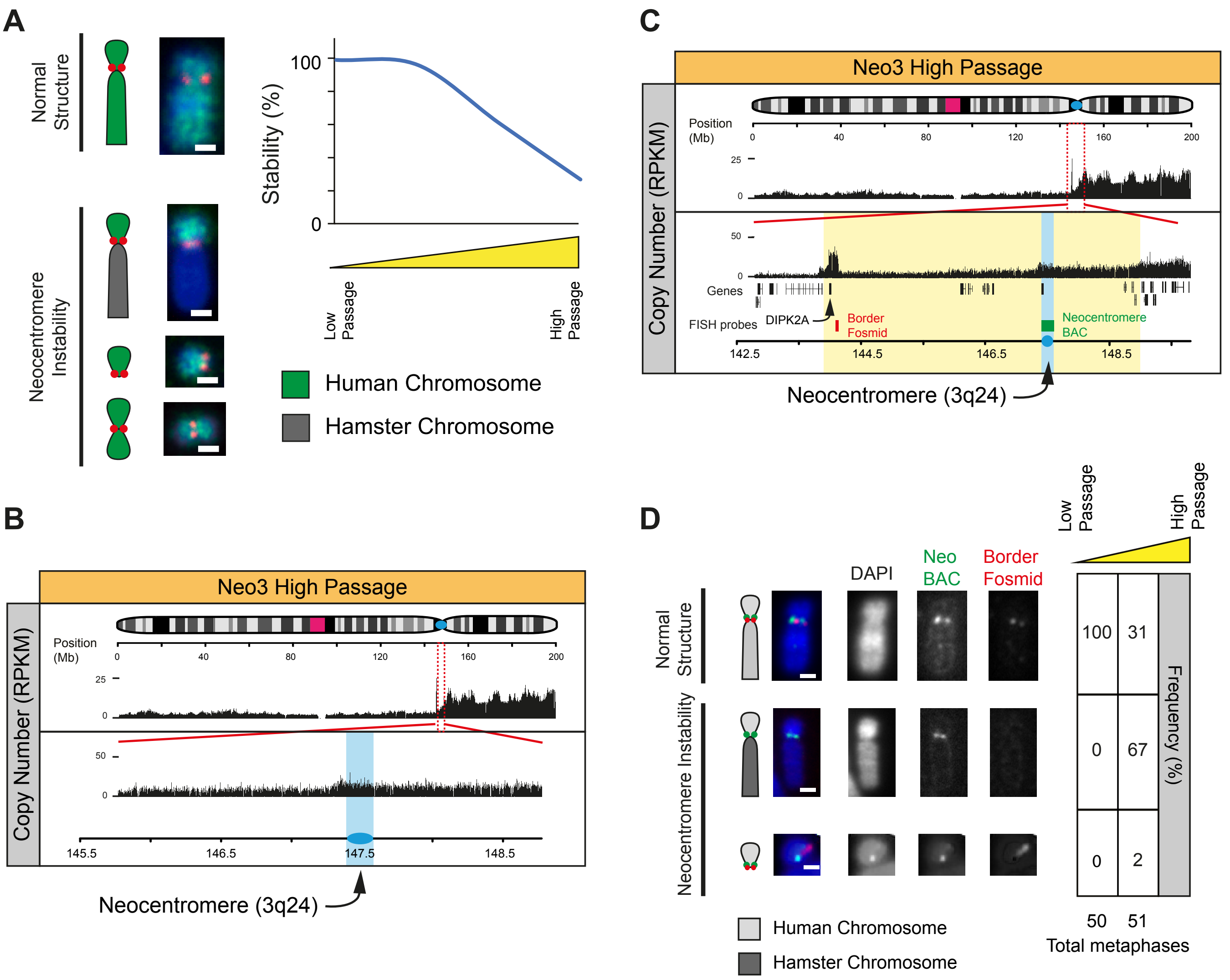

Supplementary Fig. 7. Neocentromere associated genome instability (Relates to Figure 7)
(A) Left, Representative 2D DNA FISH images of Neo3 chromosome architecture. Neo3 chromosomes were hybridized with a human chromosome 3 paint (green) and neocentromere BAC probe (red) and scored as normal or abnormal (displaying neocentromere instability in the form of breaks, fusions to hamster chromosomes (dark grey) or duplications). Right, graph quantifying loss in chromosome stability as passage number increased. Bar is 2 μm. **(B)** High passage (passage 50) Neo3 Chromosome copy number (RPKM), with a zoom in of the 3 Mb region around the neocentromere (blue). **(C)** High passage (passage 50) Neo3 Chromosome copy number (RPKM), with a zoom in of the 7 Mb region around the neocentromere (blue). Locations of DNA FISH probes are shown, neocentromere BAC probe (green) and border fosmid (red). **(D)** Left, representative FISH images of Neo3 metaphase chromosomes hybridised to a BAC (green) located at the neocentromere and a fosmid (red) located at the border. Bar is 2μm. Right, chromosome morphology was scored and quantified (%) with increasing passage number over time. Loss of the border probe signal was coincident with fusion of human Neo3 fragment (light grey) to a hamster chromosome (dark grey). Bar is 2μm.

Supplementary Table 1.

Details of the fosmid and BAC probes used for FISH to investigate large scale chromatin compaction

FISH Probe	ID	Probe location	Genomic Band	Type
G248P81751C5	A	chr3:148152154-148190706	3q24	Fosmid
G248P87211G9	B	chr3:147386613-147424083	3q24	Fosmid
G248P87849H8	C	chr3:147698288-147739058	3q24	Fosmid
G248P8432C9	D	chr3:146792070-146832279	3q24	Fosmid
G248P8026G2	G	chr3:147128170-147169864	3q24	Fosmid
RP11-1068F8	Neocentromere BAC	chr3:147402395-147564508	3q24	BAC
WI2-2366F2	Border Fosmid	chr3:144079778-144118953	3q24	Fosmid
p82H	α-satellite	centromere		
WI2-898I21	A	chr6:26248648-26287393	6p22.2	Fosmid
WI2-2980M22	C	chr6:26394868-26435139	6p22.2	Fosmid

Fosmids and BACs were obtained from BacPac resources, DNA co-ordinates are hg38

Supplementary Table 2.

Primer sequence information

Primer name	Sequence	Figure
DIPK2A_Fwd	TTTGCAGTTGGTCCTAGAGATG	Supplementary Fig. 2
DIPK2A _Rev	AAGCCTCCTTATCACAGTCATC	Supplementary Fig. 2
PLOD2_Fwd	CTGAACGACAGCGTTCTCTTC	Supplementary Fig. 2
PLOD2_Rev	CCACCTCCCTGAAAGTCTTCT	Supplementary Fig. 2
PLSCR1_Fwd	GGACAGAGGGTTTACTTTGC	Supplementary Fig. 2
PLSCR1_Rev	GGTCTCTCCAGAGTTATGAC	Supplementary Fig. 2
ZIC1_Fwd	GTCCTACACGCATCCCAGTT	Supplementary Fig. 2
ZIC1_Rev	GTGGAGGATTCGTAGCCAGA	Supplementary Fig. 2
ZIC4_Fwd	AGGCTCAAAGTCAGAAAATG	Supplementary Fig. 2
ZIC4_Rev	TAAGAGTGTTTCGGTAAAGC	Supplementary Fig. 2
HLTF_Fwd	TGAAATGGAACCAGCTGAGG	Supplementary Fig. 2
HLTF_Rev	GTATAAGTCATTTCGCTGTTCCC	Supplementary Fig. 2
DIPK2A _Exon/Intron_Fwd	CTACGCGGAGACCAAGGAC	Supplementary Fig. 5
DIPK2A_Exon/Intron_Rev	CCACTTCTCCCGACTCTGAT	Supplementary Fig. 5
HLFT_Exon/Intron_Fwd	AGCGGTTTCAGATCAGTTGA	Supplementary Fig. 5
HLFT_Exon/Intron_Rev	AGTTGTACCTTGGAGCCTTGA	Supplementary Fig. 5
