# Supplementary material for: Human centromere repositioning activates transcription and opens chromatin fibre structure
Source: Nat Commun. 2022 Sep 24;13:5609. doi: 10.1038/s41467-022-33426-2 (PMC9509383; doi:10.1038/s41467-022-33426-2)
Supplement: Supplementary file 3 — Reporting Summary [file 41467_2022_33426_MOESM3_ESM.pdf]

nature portfolio

Corresponding author(s): Nick Gilbert

Last updated by author(s): September 7th 2022

Reporting Summary

Nature Portfolio wishes to improve the reproducibility of the work that we publish. This form provides structure for consistency and transparency in reporting. For further information on Nature Portfolio policies, see our Editorial Policies and the Editorial Policy Checklist.

Statistics

For all statistical analyses, confirm that the following items are present in the figure legend, table legend, main text, or Methods section.

n/a	Confirmed	
☐	☒	The exact sample size (*n*) for each experimental group/condition, given as a discrete number and unit of measurement
☒	☐	A statement on whether measurements were taken from distinct samples or whether the same sample was measured repeatedly
☐	☒	The statistical test(s) used AND whether they are one- or two-sided<br>*Only common tests should be described solely by name; describe more complex techniques in the Methods section.*
☒	☐	A description of all covariates tested
☒	☐	A description of any assumptions or corrections, such as tests of normality and adjustment for multiple comparisons
☐	☒	A full description of the statistical parameters including central tendency (e.g. means) or other basic estimates (e.g. regression coefficient) AND variation (e.g. standard deviation) or associated estimates of uncertainty (e.g. confidence intervals)
☐	☒	For null hypothesis testing, the test statistic (e.g. *F*, *t*, *r*) with confidence intervals, effect sizes, degrees of freedom and *P* value noted<br>*Give P values as exact values whenever suitable.*
☒	☐	For Bayesian analysis, information on the choice of priors and Markov chain Monte Carlo settings
☒	☐	For hierarchical and complex designs, identification of the appropriate level for tests and full reporting of outcomes
☒	☐	Estimates of effect sizes (e.g. Cohen's *d*, Pearson's *r*), indicating how they were calculated

Our web collection on statistics for biologists contains articles on many of the points above.

Software and code

Policy information about availability of computer code

Data collection	The following software was used to collect FISH imaging data: Micromanager (Version 1.4) , Nikon Nis-Elements software (Version 4.6), Volocity (Version 6.2.1) and Imaris (Version 9.6.0). Custom 180k Agilent microarrays were designed using Agilent SureDesign www.earray.chem.agilent.com/suredesign. Microarray data was collected using Agilent Feature Extraction software (Version 11.5). DNA sequence capture probes were designed using the Nimblegencapture design software Nimbledesign (Version 4.3).
Data analysis	FISH image analysis used Volocity (version 6.2.1), Imaris (Version 9.6.0)and a custom iVision (Version 4.5.6 r4) script that calculates the distance between two fosmid probe signals. Microarray analysis in R (Version 4.2) used the RINGO Bioconductor package (Version 1.46), Bioconductor LIMMA package (Version 3.38.3) and Zoo package (Version 1.8-6). RNA-seq analysis was performed using TopHat (Version 2.1.1) and Bowtie2 (Version 2.2.6). Aligned BAM files were processed with Samtools (Version 1.6) and the Bedtools “genome coverage” tool (Version 2.25.0). TT-seq analysis was performed using Bowtie2 (Version 2.2.6) and processed with Samtools (Version 1.6), and the deepTools “bamCoverage” tool (Version 3.2.0). Shallow DNA-seq analysis was performed using BWA (Version 0.7.5a) and processed with Samtools (Version 1.6). In R (Version 4.2) the BAM files were loaded into the Bioconductor (Version 3.15) package QDNAseq (Version 1.18) for copy number analysis. Nucleosome positioning analysis used Bowtie2 (Version 2.2.6) with high quality (mq > 20) followed by the Bedtools “bamtobed” tool (Version 2.25.0) and the NucleR (Version 2.28), Zoo (Version 1.8-6) and lattice (Version 0.20.38) packages in R (Version 4.2).

For manuscripts utilizing custom algorithms or software that are central to the research but not yet described in published literature, software must be made available to editors and reviewers. We strongly encourage code deposition in a community repository (e.g. GitHub). See the Nature Portfolio guidelines for submitting code & software for further information.

Data

Policy information about availability of data

All manuscripts must include a data availability statement. This statement should provide the following information, where applicable:

- Accession codes, unique identifiers, or web links for publicly available datasets
- A description of any restrictions on data availability
- For clinical datasets or third party data, please ensure that the statement adheres to our policy

Source data are provided within this paper as a Source Data file. The data reported in this paper are publicly available on GEO: SuperSeries GSE195886. This is composed of the following SubSeries: GSE195883 (Agilent 'open' chromatin-chip), GSE195884 (Agilent bTMP-chip), GSE195885 (Agilent ChIP-chip), GSE196155 (TTseq), and GSE196160 (RNAseq). Agilent arrays were designed using human reference genome (hg19) (GSE195883, GSE195884 and GSE195885). RNAseq data (GSE196160) is aligned to human reference genome (hg19). TTseq data (GSE196155) is aligned to Human(hg38) /Hamster (GCA_003668045.1) hybrid reference genome.

Human research participants

Policy information about studies involving human research participants and Sex and Gender in Research.

Reporting on sex and gender	Not applicable.
Population characteristics	Not applicable.
Recruitment	Not applicable.
Ethics oversight	Not applicable.

Note that full information on the approval of the study protocol must also be provided in the manuscript.

Field-specific reporting

Please select the one below that is the best fit for your research. If you are not sure, read the appropriate sections before making your selection.

[x] Life sciences   [ ] Behavioural & social sciences   [ ] Ecological, evolutionary & environmental sciences

For a reference copy of the document with all sections, see nature.com/documents/nr-reporting-summary-flat.pdf

Life sciences study design

All studies must disclose on these points even when the disclosure is negative.

Sample size	No a priori sample size calculation was performed. FISH Imaging analysis sample sizes ranged from 24 - 103. Sample size varied between experiment due to variability between cell number per slide and/or visualization of fosmid probes used per individual experiment. Actual sample numbers per experiment are shown on each figure. We deemed these sample numbers to be sufficient as the experiment with the lowest number of nuclei (24) gave the same result when only half the number of nuclei were analyzed.
Data exclusions	Microarray data analysis excluded a region on chr3: 147324413-147482213 which represented a small block of GC-rich DNA that amplified aberrantly and was therefore blacklisted.
Replication	For the majority of experiments a minimum of two biological replicates was used and experimental findings were reproduced in the replicate experiment. In a small number of experiments one biological replicate was used but the result was verified by another experiment.
Randomization	This study specifically looked at a human neocentromere and therefore did not involve any experiments which would have required sample randomization. As this study focused on a specific neocentromere locus, either in the same cell or in two cell lines identically treated, there were no applicable covariates to control for.
Blinding	Blinding was taken into account for all FISH imaging experiments and analysis. FISH slides were coded so that the researcher was blind to cell line and sample treatment for both FISH imaging and analysis.

Reporting for specific materials, systems and methods

We require information from authors about some types of materials, experimental systems and methods used in many studies. Here, indicate whether each material, system or method listed is relevant to your study. If you are not sure if a list item applies to your research, read the appropriate section before selecting a response.

Materials & experimental systems

n/a	Involved in the study
☐	☒ Antibodies
☐	☒ Eukaryotic cell lines
☒	☐ Palaeontology and archaeology
☒	☐ Animals and other organisms
☒	☐ Clinical data
☒	☐ Dual use research of concern

Methods

n/a	Involved in the study
☒	☐ ChIP-seq
☒	☐ Flow cytometry
☒	☐ MRI-based neuroimaging

Antibodies

Antibodies used

Avidin FITC (Vector, A-2011); Avidin Texas Red (Vector, A-2016); Biotinylated anti-avidin (Vector, BA-0300); FITC anti-sheep (Vector, FI 6000); Texas Red anti-sheep (Vector, TI 6000), Streptavidin Cy5 (Vector, SA-1500-1); anti-RNA polymerase II (Abcam, Ab24758, clone H5); FITC anti-mouse (Jackson ImmunoResearch laboratories, 115-095-003); Texas Red anti-rabbit (Jackson ImmunoResearch laboratories, 711-075-152); CENP-A and CENP-C (non commercial, provided by provided by Stefania Purgato (author)); H3K27ac (Abcam, Ab4729); H3K4me2 (Millipore, 07-030); H3K9me2 (Millipore, 07-212); H3K9me3 (Abcam, Ab8898); H3K27me3 (Abcam, Ab6002); H4K20me1 (Abcam, Ab9051); H3K36me3 (Abcam, Ab9050); CTCF (Cell Signaling Technology, 2899); RNA Polymerase II (Diagenode, C15200004); RNA Polymerase II (gift from H. Kimura); EXOSC3 (Abcam, Ab156683).

Validation

CENP-A and CENP-C antibodies (non commercial antibodies provided by Stefania Purgato (author) are previously validated human centromere specific antibodies; Trazzi, S. et al. The C-terminal domain of CENP-C displays multiple and critical functions for mammalian centromere formation. PLoS One 4, e5832 (2009). We confirmed that both of these antibodies were centromere specific using immunocytochemistry on metaphase spreads of parental human Neo3 cells. This CENP-A antibody and several others we tried (ab13939 (Abcam), sc-11278 (Santa Cruz Biotechnology, Inc) and sc-22814 (Santa Cruz Biotechnology, Inc)) in both immunocytochemistry and ChIP, failed in the human-hamster hybrid cell lines as these antibod¬ies did not cross react well with hamster CENP-A. This is likely due to reduced sequence conservation be¬tween Human, Hamster and Mouse CENP-A (Figueroa, J., Pendón, C. & Valdivia, M. M. Molecular cloning and sequence analysis of hamster CENP-A cDNA. BMC Genomics 3, 11 (2002). The CENP-C antibody (de¬signed against aa 23-410 of human CENP-C where there is significant overlap with hamster CENP-C) was centromere specific in immunocytochemistry analysis in both GM10253A and HybNeo3 human-hamster hybrid cell lines cell lines. These antibodies were additionally validated by western blot and ChIP was optimised by ChIP-qPCR.

RNA Polymerase II (Diagenode, C15200004): The datasheet for this antibody confirms species reactivity in Human, Xenopus and Yeast and states other species were not tested. We validated this antibody by western blot in the human-hamster hybrid cell lines and ChIP was optimised by ChIP-qPCR.

RNA polymerase II (Abcam, Ab24758, clone H5): The datasheet for this antibody confirms species reactivity in Human, Mouse, Zebrafish and Drosophila melanogaster. We validated this antibody by western blot and immunofluorescence (Fig. 2C) in human-hamster hybrid cell lines.

RNA Polymerase II (gift from H. Kimura): http://antibodyregistry.org/AB_2827956
Stasevich et al. Regulation of RNA polymerase II activation by histone acetylation in single living cells. Nature. 2014 Dec 11;516 (7530):272-5 This antibody was designed against the target antigen of human RNA polymerase II C-terminal domain (CTD) and also detects mouse RNA polymerase. We validated this antibody by western blot in the human-hamster hybrid cell lines and ChIP was optimised by ChIP-qPCR.

H3K27ac (Abcam, Ab4729): The datasheet for this antibody confirms species reactivity in Mouse, Rat, Cow, Human and Recombinant fragment. It is predicted to work with: Chicken, Xenopus laevis, Arabidopsis thaliana, Drosophila melanogaster, Monkey, Zebrafish, Plasmodium falciparum, Rice, and Cyanidioschyzon merolae. We validated this antibody by western blot in the human-hamster hybrid cell lines and ChIP was optimised by ChIP-qPCR.

H3K4me2 (Millipore, 07-030): The datasheet for this antibody confirms species reactivity in Human and tetrahymena and also states that broad species cross-reactivity is expected. We validated this antibody by western blot in the human-hamster hybrid cell lines and ChIP was optimised by ChIP-qPCR.

H3K9me2 (Millipore, 07-212): The datasheet for this antibody confirms species reactivity in Human, Chicken, Vertebrates and Yeast. We validated this antibody by western blot in the human-hamster hybrid cell lines and ChIP was optimised by ChIP-qPCR.

H3K9me3 (Abcam, Ab8898): The datasheet for this antibody confirms species reactivity in Mouse, Cow and Human. It is predicted to work with Rat, Chicken, Saccharomyces cerevisiae, Xenopus laevis, Drosophila melanogaster, Indian muntjac, Mammals, Xenopus tropicalis and Cyanidioschyzon merolae. We validated this antibody by western blot in the human-hamster hybrid cell lines and ChIP was optimised by ChIP-qPCR.

H3K27me3 (Abcam, Ab6002): The datasheet for this antibody confirms species reactivity in Mouse, Cow, Human and Recombinant fragments. It is predicted to work with Rat, Rabbit, Chicken, Xenopus laevis, Arabidopsis thaliana, Drosophila melanogaster, Plants, Zebrafish, Rhesus monkey, Chinese hamster and Rice. We validated this antibody by western blot in the human-hamster hybrid cell lines and ChIP was optimised by ChIP-qPCR.

H3K36me3 (Abcam, Ab9050): The datasheet for this antibody confirms species reactivity in Cow and Human. It is predicted to work

with Mouse, Rat, Saccharomyces cerevisiae, Xenopus laevis, Arabidopsis thaliana, Caenorhabditis elegans, Drosophila melanogaster, Plants, Schizosaccharomyces pombe, Zebrafish, Silk worm, Rice, Xenopus tropicalis, Trypanosoma brucei. We validated this antibody by western blot in the human-hamster hybrid cell lines and ChIP was optimised by ChIP-qPCR.

H4K20me1 (Abcam, Ab9051): The datasheet for this antibody confirms species reactivity in Mouse, Cow, Human, Caenorhabditis elegans, Drosophila melanogaster and Schizosaccharomyces pombe. It is predicted to work with Mammals. We validated this antibody by western blot in the human-hamster hybrid cell lines and ChIP was optimised by ChIP-qPCR.

CTCF (Cell Signaling Technology, 2899): The datasheet for this antibody confirms species reactivity in Human, Mouse, Rat and Monkey. We validated this antibody by western blot in the human-hamster hybrid cell lines and ChIP was optimised by ChIP-qPCR.

EXOSC3 (Abcam, Ab156683): The datasheet for this antibody confirms species reactivity in Mouse and Human. It is predicted to work with Chimpanzee, Macaque monkey, Rhesus monkey, Gorilla, Common marmoset and Orangutan. We validated this antibody by western blot in the human-hamster hybrid cell lines (Fig. 2D).

Eukaryotic cell lines

Policy information about cell lines and Sex and Gender in Research

Cell line source(s)	Human parental lymphoblastoid cell lines Neo3 and Neo6 and Human-Hamster hybrid cell lines GM10253A and HybNeo3 provided by Mariano Rocchi (author).
Authentication	This study used unique cell lines where authentication is not available.
Mycoplasma contamination	All cell lines tested negative for Mycoplasma.
Commonly misidentified lines (See ICLAC register)	No misidentified cell lines were used.
